# First Draft Genome Assembly of Root-Lesion Nematode *Pratylenchus scribneri* Generated Using Long-Read Sequencing

**DOI:** 10.3390/ijms24087311

**Published:** 2023-04-15

**Authors:** Deepika Arora, Alvaro G. Hernandez, Kimberly K. O. Walden, Christopher J. Fields, Guiping Yan

**Affiliations:** 1 Department of Plant Pathology, North Dakota State University, Fargo, ND 58105, USA; deepika.arora@ndus.edu; 2 Roy J. Carver Biotechnology Center, University of Illinois at Urbana-Champaign, Urbana, IL 61820, USA; aghernan@illinois.edu (A.G.H.); kosterbu@illinois.edu (K.K.O.W.); cjfields@illinois.edu (C.J.F.)

**Keywords:** root-lesion nematode, genome, ultra-low DNA input, contigs, sequencing, BUSCO, diploid

## Abstract

Root-lesion nematodes (genus *Pratylenchus*) belong to a diverse group of plant-parasitic nematodes (PPN) with a worldwide distribution. Despite being an economically important PPN group of more than 100 species, genome information related to *Pratylenchus* genus is scarcely available. Here, we report the draft genome assembly of *Pratylenchus scribneri* generated on the PacBio Sequel IIe System using the ultra-low DNA input HiFi sequencing workflow. The final assembly created using 500 nematodes consisted of 276 decontaminated contigs, with an average contig N50 of 1.72 Mb and an assembled draft genome size of 227.24 Mb consisting of 51,146 predicted protein sequences. The benchmarking universal single-copy ortholog (BUSCO) analysis with 3131 nematode BUSCO groups indicated that 65.4% of the BUSCOs were complete, whereas 24.0%, 41.4%, and 1.8% were single-copy, duplicated, and fragmented, respectively, and 32.8% were missing. The outputs from GenomeScope2 and Smudgeplots converged towards a diploid genome for *P. scribneri*. The data provided here will facilitate future studies on host plant-nematode interactions and crop protection at the molecular level.

## 1. Introduction

Root-lesion nematodes (RLN) are recognized as the top three plant-parasitic nematode groups of the world and lie behind root-knot (*Meloidogyne*) and cyst nematodes (*Heterodera*) in terms of economic importance [1]. Unlike sedentary endoparasites (*Meloidogyne* and *Heterodera*), nematodes in the genus *Pratylenchus* do not establish permanent feeding sites (giant cells or syncytia) and move freely between soil and roots throughout their lifecycle. This feeding behavior results in the development of characteristic reddish-brown necrotic lesions on root tissue, which construct pathways for secondary invaders, such as bacteria, fungi, etc., resulting in severe yield losses [1,2]. *Pratylenchus* spp. have a cosmopolitan distribution and are known to parasitize more than 400 host plant species. They have a wide host range and impact crops of major economic importance, such as potato, wheat, corn, and soybean [3]. The use of chemical nematicides is a common management strategy for controlling RLNs, but their toxic effects on non-target organisms and environmental health call for alternative control strategies based on novel gene targets [4].

The root-lesion nematode, *Pratylenchus scribneri* Steiner 1943, is found commonly associated with the major crops grown in the northern Great Plains region of North America [5,6,7]. Considering the importance of *P. scribneri*, several molecular detection methods for identifying and quantifying this nematode in soil and roots have been developed to enhance the management decisions and practices [8,9,10]. More recently, it has been reported as an important nematode pest infecting corn, soybean, wheat, and tomato in China [11,12,13,14]. In Nigeria, yield losses in corn have been reported to be between 26 to 37% based on the population densities of *P. scribneri* [15].

Despite the ubiquity and significant importance of *Pratylenchus* species in agriculture, the availability of genome information on these species is limited as compared to the sedentary PPN. So far, the genome sequence of only one *Pratylenchus* species, *P. coffeae*, is available, and the published genome size of 19.7 Mb is the smallest among the metazoans [16]. This raises questions regarding its entirety, especially due to the lack of a complete physical map. Additionally, the genomes *of Pratylenchus* spp. are expected to have complex ploidy similar to root-knot nematodes (*Meloidogyne* spp.) [17], but this is still an unexplored area. Ploidy determination could provide important insights into the developmental and evolutionary biology of *Pratylenchus* spp. Moreover, the genome data for different *Pratylenchus* spp. are needed to help better understand the parasitism mechanism of the nematodes in this genus. Jointly, this information could help reveal novel gene targets for employing specific control strategies for *P. scribneri* and other related species. In light of this, the main objective of the current study is to generate a contiguous and relatively complete annotated genome of one of the important root-lesion nematodes, *P. scribneri*, and make it publicly available. We also explored two different genomic DNA isolation methods to prepare sequencing quality DNA for *P. scribneri*, and the advantages of one over the other have been presented.

## 2. Results

### 2.1. Library Preparation and Assembly

The raw PacBio HiFi sequencing yields and assembly metrics for the libraries prepared using two different DNA isolation methods are presented in detail in Table 1. The library resulting from DNA extracted using 500 handpicked nematodes (Method 2) had greater sequencing yield (19.3 Gb) as compared to Method 1 (14.9 Gb), which involved DNA isolation directly from nematode suspension. Fewer contigs were assembled in Method 2. The contig N50 increased from 222 kb in the first method to 1.70 Mb in the second method. The average GC% was 37.6% and 32.8% for sequencing libraries prepared using Method 1 and Method 2, respectively.

### 2.2. Assembly Decontamination and Completeness Analysis

**Figure 1:**
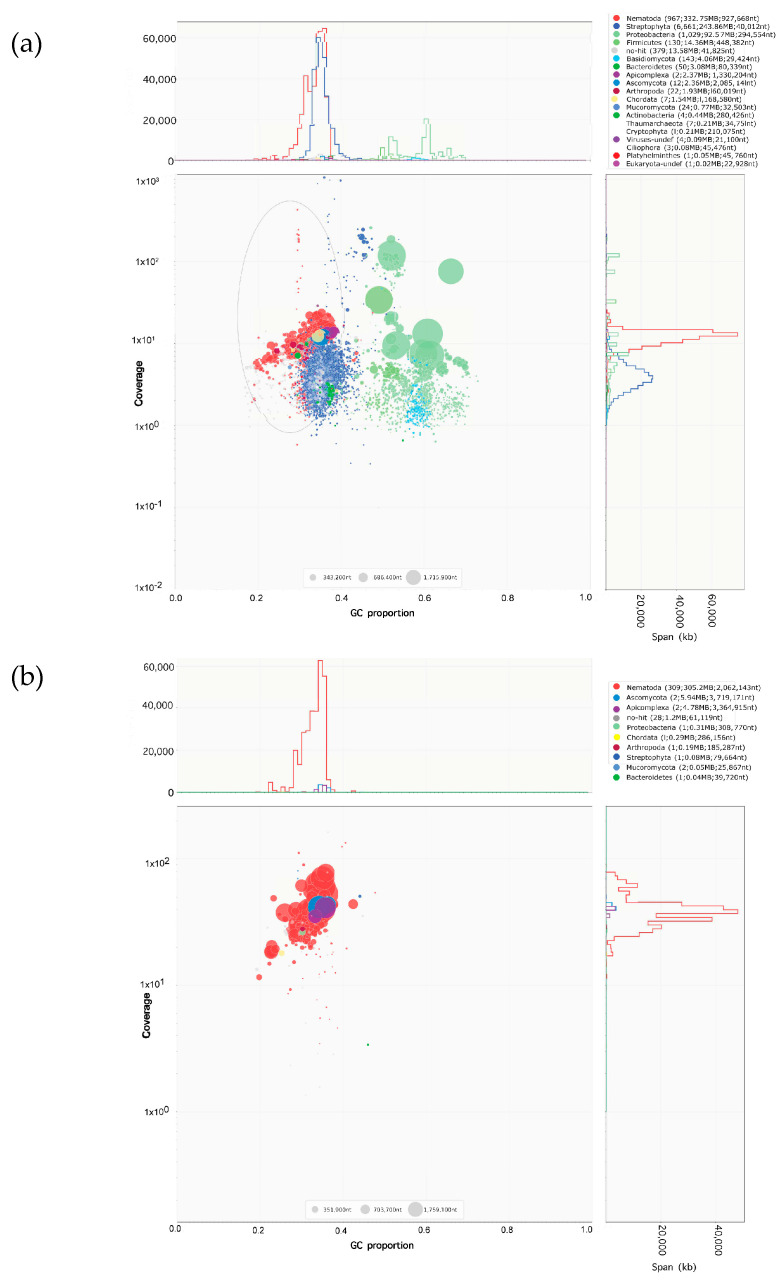
Blobplot contaminant analysis of *Pratylenchus scribneri* assembly obtained using two DNA extraction methods, before removing contaminants. The contig length is represented by circles proportionally scaled and colored by taxonomic annotation based on BLAST similarity search results. Nematode genome contigs are represented by red circles for: (**a**) Method 1 (library prepared from 8000 to 10,000 mixed-stage *P. scribneri* in nematode suspension obtained from carrot culture); (**b**) Method 2 (library prepared from 500 hand-picked *P. scribneri* juveniles and adults). Contigs are placed based on the GC proportion (*X*-axis) and the coverage of reads (*Y*-axis). The legend box on the top right of each Blobplot provides a color-coded description of the different taxonomic groups identified.

The Blobplot in Figure 1 represents the preliminary contig taxonomic assignments, contig read coverage, contig lengths, and contig GC% obtained for the two DNA extraction methods. Figure 1a,b show the Blobplot analyses from the “core” nematode genome contigs as red points, and that the library constructed using Method 2 showed >30× *P. scribneri* read coverage than the library constructed using Method 1. In Method 2, out of the 39 contigs classified into phyla other than Nematoda, only one contig was determined to be a bacterial contaminant sequence which was removed prior to further genome analysis. All others had very weak hits to a variety of phyla tested or no hits at all. The assembly obtained from Method 2 was used for further analyses and subject to haplotig purging. The final purged decontaminated assembly contained 276 contigs summing to ~227 Mb. The BUSCO scores for raw, purged, and decontaminated assembly are provided in Table 2. The final decontaminated assembly had a completeness score of 65.4%, with 24.0% being single copy, 41.4% duplicated, 1.8% fragmented, and 32.8% missing BUSCOs out of the total 3131 orthologs used.

### 2.3. Genome Ploidy Estimation and Structural Annotation

**Figure 2:**
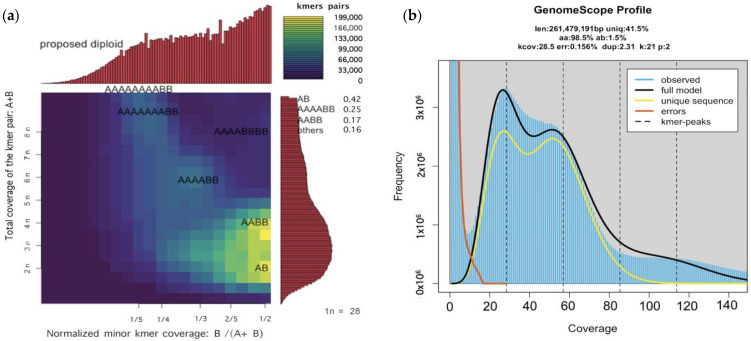
Genome ploidy estimation of *Pratylenchus scribneri* genome assembly: (**a**) Smudgeplots showing the coverage and distribution of k-mer pairs that fit to diploid genome model; (**b**) GenomeScope k-mer (k = 21) profile plot of the *P. scribneri* showing the fit of the GenomeScope model (black) to the observed k-mer frequencies (blue) with two major peaks.

The *P. scribneri* genome ploidy estimations were made using the final assembly constructed using Method 2. After manually setting the estimated monoploid peak from GenomeScope and Smudgeplot, the models and output converged on ploidy = 2. The presence of two major peaks in GenomeScope output and coverage and distribution of k-mer pairs into two bright smudges in Smudgeplot pointed towards a diploid genome for *P. scribneri* (Figure 2).

For predicting proteins, the BUSCO analysis was run in “long mode” on the final decontaminated assembly. The predicted proteins (single copy (23.5%)/multi-copy (42.7%)/fragmented (1.4%)) were then merged together into 3966 protein sequences as additional protein input for BRAKER annotation. The structural annotation ran using BRAKER and TSERBA with RNA-seq and protein evidence resulted in a total of 51,146 predicted protein sequences. The BUSCO scores for the TSEBRA-combined BRAKER2 predicted protein had 72% complete BUSCOs (single copy: 12.0%, duplicate: 60.0%, fragmented: 1.2%) and 26.8% missing BUSCOs.

## 3. Discussion

Recently, long-read PacBio HiFi sequencing has emerged as a powerful tool to generate high-quality de novo genome assemblies. However, the technique has not been explored to its full potential for microorganisms such as nematodes, due to the relatively high DNA input requiring a larger number of nematodes. Here, we present, for the first time, the de novo draft genome assembly of the root-lesion nematode *Pratylenchus scribneri* constructed using 5 ng of input DNA from 500 nematodes as an example. In this study, the libraries prepared from two different DNA extraction methods were sequenced using the PacBio ultra-low DNA input protocol which uses as low as 5 ng of DNA as input and therefore, significantly fewer nematodes. The library prepared using DNA extracted from 500 handpicked nematodes (Method 2) was much cleaner (fewer contaminants) as compared to the library prepared from DNA extracted directly from nematode suspension (Method 1). This could be attributed to the presence of carrot tissue and other organisms in the library prepared using the nematode suspension obtained directly from carrot cultures. As a result, the number of assembled contigs specific to different taxonomical groups were reduced from 9.5 k in Method 1 to 492 in Method 2. It is difficult to get the sequencing library from nematodes grown on carrots to be completely free of any contaminants. For optimal results, the handpicking of nematodes required for Method 2 should be carried out in a laminar flow hood under sterile conditions, but Method 2 is also tedious due to the handpicking of 500 nematodes, which could be a limitation for preparing the nematode sample for DNA extraction.

There are only a few reports available on the de novo genome assembly construction of small organisms based on the ultra-low DNA input protocol. Kingan et al. [18] generated a high-quality de novo genome assembly using a single mosquito with the ultra-low DNA protocol. DNA isolation for this study was performed using the MagAttract HMW kit as used in Method 2 for the current study. However, no reports on de novo genome assembly of a plant-parasitic nematode using the ultra-low DNA input protocol are available thus far. The use of MagAttract HMW kit for genomic DNA isolation for whole genome sequencing has mostly been observed for bacterial pathogens and insects [18,19,20]. In the present study, the library prepared using 5 ng of input DNA from 500 active *P. scribneri* individuals (Method 2) was sequenced with a single PacBio SMRT cell 8 M chip on the Sequel IIe System. Nearly 2.7 million reads producing 19.3 Gb of raw data with mean read length of 7 kb were obtained and the genome size was predicted to be around 227 Mb. Similar raw sequencing yield results were obtained for the sand fly (*Phlebotomus papatasi*) sequenced on the Sequel IIe System using just 5 ng of input DNA following PacBio’s ultralow DNA input protocol (https://www.pacb.com/wp-content/uploads/Korlach-PAG-2020-High-Quality-PacBio-Insect-Genome-from-5-ng-of-Input-DNA.pdf, accessed on 30 November 2022).

The BUSCO completeness scores for the final decontaminated assembly tested against 3131 orthologs were fairly average and the presence of more than 65% complete genes in our study are comparable with previous studies [21,22]. However, the gene duplication content of our assembly was higher, and could be related to the likely repetitive gene content of *P. scribneri* genome and usage of orthologs from different reference datasets in other studies on PPNs [19,22]. This in part may also explain the presence of fewer complete and duplicated BUSCOs in genome assemblies of other migratory endoparasitic nematodes such as *Radopholus similis* and *Bursaphelenchus xylophilus* [22,23]. Additionally, the genome assembly of *P. scribneri* generated in this study had a GC content of 32.8%, which is consistent with other PPNs sequenced on a PacBio RSII platform [24,25]. However, the completeness and quality of the set of 51,146 predicted proteins in *P. scribneri* assessed using BUSCO showed 72% completeness. The BUSCO scores for predicted proteins were improved over the genome assembly; however, duplicated complete genes were still high (60%). Similar observations related to higher duplicated genes in the protein set of a root-knot nematode *Meloidogyne enterolobii* were noted by Koutsovoulos et al. [26]. The 227.2 Mb genome assembly size of *P. scribneri* is much bigger than 19.6 Mb of *P. coffeae*, the only *Pratylenchus* species sequenced and reported so far [16]. The above observation is supported by the underlying genome organization of *P. scribneri* containing a fair amount of gene duplication. This study suggests a diploid genome for *P. scribneri* which is consistent with ploidy estimations of another migratory endoparasitic nematode, *B. xylophilus* [27]. Further studies on comparative and functional genomics analysis are needed to validate these results and take a deeper look into the common genes and their functions between the two species.

To the best of our knowledge, this is the first report of de novo draft assembly of the *P. scribneri* genome generated using the PacBio ultra-low DNA input protocol. The new workflow described here will facilitate the sequencing and assembly of new and existing species of plant-parasitic nematodes that are important for crop production. In vitro culturing of nematodes is a common technique to increase pure nematode population. The DNA preparation and sequencing method described here is recommended for sequencing genome of the nematodes that are difficult to be mass-produced. Overall, the strategy of hand-picking nematodes under sterile conditions performed better in our study and resulted in an almost contaminant free library and can be considered for other endoparasitic and ectoparasitic plant-parasitic nematodes. The long-read-based sequencing and genome assembly of *P. scribneri* would enable the identification of parasitism genes/effectors involved in the host and nematode interaction mechanism. Additionally, the comparative genome analysis of *Pratylenchus* species with that of sedentary endoparasites could facilitate studies associated with evolutionary and lifestyle mechanisms of plant-parasitic nematodes based on the effectors present in different groups.

## 4. Materials and Methods

### 4.1. Nematode Collection, DNA Isolation, and Evaluation

*Pratylenchus scribneri* isolate was originally collected from a potato field (in rotation with corn) located in Sargent County, ND and, thereafter, maintained aseptically on carrot discs in our lab as suggested by Lawn and Noel [28]. For preparing nematode suspension, the inoculated carrot discs in the Petri plates were cut into thin slices using a sterile razor blade. The carrot pieces were left suspended in water for 3 h for the nematodes to move out of the carrot tissues. The nematodes along with the carrot tissues were then passed through a 60-mesh sieve placed on a 635-mesh sieve. The nematodes retained on the 635-mesh sieve were later collected into a 50 mL tube using a wash bottle containing distilled water with 50 mg/L carbenicillin and 50 mg/L kanamycin. Starting with nematode suspension, two different methods were used to isolate *P. scribneri* genomic DNA. In the first method, a 1 mL nematode suspension containing 8000 to 10,000 mixed-stage individuals of *P. scribneri* was ground to a fine powder in liquid nitrogen. The crushed nematodes were then subject to DNA extraction using the DNAeasy Plant Mini Kit (Qiagen, Hilden, Germany). The DNA quality was assessed using a ND-1000 Nanodrop spectrophotometer (Thermo Scientific, Waltham, MA, USA) and the samples with 260/280 above 1.8 were selected for further analyses (Method 1). For the second method, 500 *P. scribneri* adults and juveniles from the original nematode suspension were handpicked and transferred under a microscope on a sterile glass slide containing 100 µL double-distilled water with antibiotics (50 mg/L carbenicillin and 50 mg/L kanamycin). Using a 200 µL pipette, the nematodes were then transferred to a 1.5 mL tube and centrifuged at 4000 rpm at 4 °C for 10 min. The nematode pellet was snap frozen in liquid nitrogen and stored in a −80 °C freezer (Method 2). For subsequent DNA extraction and sequencing, the frozen sample was sent off to the Roy J. Carver Biotechnology Center, University of Illinois at Urbana-Champaign on dry ice through overnight shipping. The nematode pellet was then pulverized under liquid nitrogen. Two hundred microliters of CTAB (OPS Diagnostics, Lebanon, NJ, USA) were added, mixed with the powder, and the sample was incubated at 60 °C for 60 min and cooled to room temperature. The MagAttract HMW DNA (Qiagen, Hilden, Germany) kit was used for the rest of the procedure, with some modifications. Briefly, proteinase K and RNAase were added to the mixture and incubated overnight at room temperature. The mixture was cleaned up with 1× volume of chloroform/isoamyl alcohol, gently inverted 10 times to mix, and incubated at room temperature for 5 min, followed by centrifugation for 2 min at 10,000× *g*. The supernatant was transferred to a new 1.5 mL tube, and magnetics beads from the MagAttract kit were added, washed twice, and the DNA was eluted from the beads in AE buffer at 37 °C for 30 min. The DNA was quantitated with Qubit using the High-Sensitivity dsDNA kit (ThermoFisher Scientific, Waltham, MA, USA) and the integrity was evaluated in a Femto Pulse system (Agilent, Santa Clara, CA, USA).

### 4.2. Library Preparation and Sequencing

Five nanograms of purified genomic DNA were sheared with Megaruptor 3 (Diagenode, Denville, NJ, USA) to an average size of 10 kb. The sheared gDNA was amplified using the PacBio Ultra-Low DNA Input kit following the manufacture’s recommendations (Pacific Biosciences, Menlo Park, CA, USA). The amplified gDNA was converted into a PacBio library with the SMRTbell Express Template Prep kit version 2.0 (Pacific Biosciences, Menlo Park, CA, USA). Briefly, the sheared genomic DNA was first added to an enzymatic reaction to remove single-stranded overhangs followed by treatment with repair enzymes to repair the DNA ends. The ends of the repaired, double-stranded fragments were then tailed with an A-overhang. After that, the T-overhang SMRTbell adapters were ligated and the SMRTbell library was purified with two rounds of AMPure PB bead clean up (Pacific Biosciences, Menlo Park, CA, USA). The library was quantitated with Qubit and then run on a Femto Pulse to confirm the presence of DNA fragments of the expected size. The library was sequenced on one SMRT cell 8 M on a PacBio Sequel IIe system with 30 h movie time. The circular consensus analysis was performed in real time in the instrument with SMRT Link V10.1 software (Pacific Biosciences, Menlo Park, CA, USA) using default parameters, which include 3 minimum passes and a minimum accuracy of 99%.

### 4.3. Genome Assembly, Decontamination, and Completeness Analysis

Raw PacBio HiFi reads were filtered with Seqkit v2.0.0 [29] to remove reads < 5 kb. Filtered reads were assembled with Hifiasm-v0.16.1 [30], turning off the parameter for automatic internal haplotig purging, and the GFA data output was converted to FASTA format with Gfatools v0.4 (https://github.com/lh3/gfatools, accessed on 25 April 2022). Primary contig assembly contamination by non-nematode sequences was assessed using Blobtools v1.1.1 [31]. Read depth was calculated by aligning the filtered PacBio HiFi reads to the primary contig assembly with Minimap v2.21 [32] and processing the bam file with SAMtools v1.12 [33]. Contigs were taxonomically assigned to phyla using Diamond v2.0.9 blastx [34] with the long reads option and e-value limit of 1 × 10^−25^ against the UniProt Reference Clusters UniRef100 dataset [35]. Thirty-nine contigs classified into phyla other than Nematoda were then further analyzed manually by BLASTN/BLASTX against the NCBI non-redundant (nr) datasets. The remaining primary contigs were then purged of haplotypic and duplicate content using the purge_dups v.1.2.5 tool [36]. The ortholog completeness was evaluated with benchmarking universal single-copy ortholog (BUSCO) v4.1.4 [37] using the nematoda_odb10 lineage dataset.

### 4.4. Genome Ploidy Estimations

To estimate the ploidy level of the genome, KMC v3.1.1 [38] was used to count k-mers (k = 21) in the filtered PacBio HiFi reads (>5 kb). The k-mer histogram was analyzed by GenomeScope 2 [39] with ploidy tested from *p* = 2 to *p* = 6. Haplotypic and duplicate genome content were also analyzed using heterozygous kmer pairs with the tool Smudgeplot [34].

### 4.5. Genome Structural Annotation

RepeatMasker v4.1.2 (http://www.repeatmasker.org, accessed on 5 July 2022) was used to soft-mask repeats from the final purged and decontaminated genome assembly file. Two separate runs of BRAKER2 [40] used RNA-seq and protein evidence with default parameters. The raw *P. scribneri* RNA-seq reads (read length = 100 bp) used for the above analysis were generated in our previous study through paired-end sequencing of cDNA libraries prepared from good quality total RNA extracts of several thousand pre-parasitic and parasitic nematodes on an Illumina NovaSeq 6000 sequencer (unpublished work). The reads were trimmed with Fastp v0.20.0 [41] and then aligned to the masked genome assembly using STAR v.2.7.10a [42]. Protein evidence was provided using NCBI’s Nematoda RefSeq dataset containing 18,060 protein sequences. In addition, BUSCO v5.3.2 running in “long mode” [43] was used to predict protein sequences using the 3131 “lineage nematoda_odb10” orthologs from the final purged/decontaminated/unmasked genome assembly file. The outputs from the two independent BRAKER runs were then combined with the tool TSEBRA v.1.0.3 [38] to obtain GTF and amino acid formats using Cufflinks v2.2.1 [44].

## Figures and Tables

**Table 1:** Sequencing yield and assembly metrics for *Pratylenchus scribneri* raw hifiasm libraries constructed using two different DNA isolation methods.

Sequencing Yield	Method 1 ^a^	Method 2 ^b^
Total Number of Bases (bp)	14,985,187,925	19,351,261,593
Total Number of Reads	1,665,292	2,749,471
Maximum Read Length (bp)	42,009	43,956
Minimum Read Length (bp)	295	68
Mean Read Length (bp)	8999	7038
**Assembly Metrics**		
Number of Contigs	9447	492
Total number of bp	714,324,379	361,758,083
Shortest (bp)	5119	5897
Longest (bp)	6,863,581	6,221,503
Average length (bp)	75,613	735,280
Median (bp)	30,718	334,760
Average GC%	37.60%	32.80%
Non-ACGT bases	0	0
Contig N50 (bp)	222,427	1,701,422

^a^ 8000 to 10,000 *P. scribneri* directly from nematode suspension harvested from carrot cultures used for DNA extraction using the DNAeasy Plant Mini Kit. ^b^ 500 *P. scribneri* juveniles and adults handpicked and DNA extracted using the MagAttract HMW DNA kit.

**Table 2:** Genome summary statistics and benchmarking universal single-copy ortholog (BUSCO) score estimation for raw, purged, and decontaminated *Pratylenchus scribneri* genome assemblies obtained using DNA extraction Method 2.

BUSCO Category	Raw Assembly	Purged Assembly ^a^	Decontaminated Assembly ^b^
Complete (C)	2084 ^c^ (66.5%) ^d^	2048 (65.4%)	2047 (65.4%)
Complete and single copy (S)	530 (16.9%)	753 (24.0%)	752 (24.0%)
Complete and duplicated (D)	1554 (49.6%)	1295 (41.4%)	1296 (41.4%)
Fragmented (F)	36 (1.1%)	57 (1.8%)	56 (1.8%)
Missing (M)	1011 (32.3%)	1026 (32.8%)	1025 (32.7%)
Total BUSCO groups searched	3131	3131	3131

^a^ Assembly with haplotypic and duplicate content removed from the primary set of contigs with purge_dups v.1.2.5 using default parameters. ^b^ Purged assembly with a single 40 kb contig determined to be a bacterial contaminant sequence removed. ^c^ Number of single-copy orthologues represented in each category out of the total BUSCO groups searched in Nematoda lineage (Nematoda_odb10). ^d^ Percentage of single-copy orthologues versus total BUSCO groups searched in Nematoda lineage (3131) for each category.

## Data Availability

All the raw PacBio sequence data supporting the results of this article as well as the genome assembly have been deposited at the NCBI with the following accession numbers: BioProject: PRJNA932437; BioSample: SAMN33192627; Genome Assembly: JAQSEB000000000; SRA raw data: SRP421680/SRS16720223/SRX19322091/SRR23381582. The data will be made publicly available with the published version of this manuscript.

## References

[1] Jones J.T., Haegeman A., Danchin E.G.J., Gaur H.S., Helder J., Jones M.G.K., Kikuchi T., Manzanilla-López R., Palomares-Rius J.E., Wesemael W.M.L. (2013). Top 10 plant-parasitic nematodes in molecular plant pathology. Mol. Plant Pathol..

[2] Smiley R.W. Multiplication of *Pratylenchus neglectus* and *P. thornei* on plants other than cereals. Proceedings of the Fifth International Cereal Nematode Initiative Workshop.

[3] Castillo P., Vovlas N. (2007). *Pratylenchus* (Nematoda: Pratylenchidae): Diagnosis, biology, pathogenicity and management. Nematol. Monogr. Perspect..

[4] Grynberg P., Coiti Togawa R., Dias de Freitas L., Antonino J.D., Rancurel C., Mota do Carmo Costa M., Grossi-de-Sa M.F., Miller R.N., Brasileiro A.C.M., Messenberg Guimaraes P. (2020). Comparative genomics reveals novel target genes towards specific control of plant-parasitic nematodes. Genes.

[5] Akhter N. (2019). Molecular Characterization of Root-Lesion Nematode Species from Corn Fields in North Dakota and Evaluation of Resistance in Corn Hybrids. Master’s Thesis.

[6] Ozbayrak M. (2019). DNA Barcoding of *Pratylenchus* from Agroecosystems in the Northern Great Plains of North America. Master’s Thesis.

[7] Yan G.P., Plaisance A., Huang D., Liu Z., Chapara V., Handoo Z.A. (2016). First report of the root-lesion nematode *Pratylenchus scribneri* infecting potato in North Dakota. Plant Dis..

[8] Arora D., Yan G.P., Baidoo R. (2020). Developing a real-time PCR assay for direct detection and quantification of *Pratylenchus scribneri* in field soil. Nematology.

[9] Arora D., Yan G.P. (2022). Early detection and temporal dynamics of *Pratylenchus scribneri* infection in potato roots determined using quantitative PCR and root staining. Phytopathology.

[10] Huang D., Yan G.P. (2017). Specific detection of the root-lesion nematode *Pratylenchus scribneri* using conventional and real-time PCR. Plant Dis..

[11] Li Y., Lu Q.S., Wang S., Liu Y.K., Wang K., Yuan H.X., Li H.L. (2019). Discovery of a root-lesion nematode, *Pratylenchus scribneri*, infecting corn in inner Mongolia, China. Plant Dis..

[12] Li Y., Lu Q.S., Wang S., Guo F., Xia Y.H., Wang K., Li H.L. (2019). Occurrence of *Pratylenchus scribneri* on soybean in Henan Province, China. Plant Dis..

[13] Xia Y., Li J., Hao P., Wang K., Lei B., Li H.L., Li Y.U. (2022). Discovery of root-lesion nematode (*Pratylenchus scribneri*) on corn in Hainan Province of China. Plant Dis..

[14] Xia Y.H., Li J., Sun M.R., Lei B., Li H.L., Li Y., Wang K. (2022). Identification and pathogenicity of *Pratylenchus scribneri* on tomato in Sichuan Province of People’s Republic of China. J. Helminthol..

[15] Olowe T. (2011). Relationship between inoculum density levels of *Pratylenchus scribneri*, and growth and yield of maize, *Zea mays*. Int. J. Nematol..

[16] Burke M., Scholl E.H., Bird D.M., Schaff J.E., Colman S.D., Crowell R., Diener S., Gordon O., Graham S., Wang X. (2015). The plant parasite *Pratylenchus coffeae* carries a minimal nematode genome. Nematology.

[17] Fosu-Nyarko J., Jones M.G.K. (2016). Advances in understanding the molecular mechanisms of root lesion nematode host interactions. Annu. Rev. Phytopathol..

[18] Kingan S.B., Heaton H., Cudini J., Lambert C.C., Baybayan P., Galvin B.D., Durbin R., Korlach J., Lawniczak M.K. (2019). A high-quality de novo genome assembly from a single mosquito using PacBio sequencing. Genes.

[19] Rufai S.B., McIntosh F., Poojary I., Chothe S., Sebastian A., Albert I., Praul C., Venkatesan M., Mahata G., Maity H. (2021). Complete Genome Sequence of *Mycobacterium orygis* Strain 51145. Microbiol. Resour. Announc..

[20] Crowley L.M. (2021). Darwin Tree of Life Consortium. The genome sequence of the seven-spotted ladybird, *Coccinella septempunctata* Linnaeus, 1758. Wellcome Open Res..

[21] Bali S., Hu S., Vining K., Brown C., Mojtahedi H., Zhang L., Gleason C., Sathuvalli V. (2021). Nematode Genome Announcement: Draft genome of *Meloidogyne chitwoodi*, an economically important pest of potato in the Pacific Northwest. Mol. Plant-Microbe Interact..

[22] Wram C.L., Hesse C.N., Wasala S.K., Howe D.K., Peetz A.B., Denver D.R., Humphreys-Pereira D., Zasada I.A. (2019). Genome announcement: The draft genomes of two *Radopholus similis* populations from Costa Rica. J. Nematol..

[23] Ding X., Guo Y., Ye J., Wu X., Lin S., Chen F., Zhu L., Huang L., Song X., Zhang Y. (2022). Population differentiation and epidemic tracking of *Bursaphelenchus xylophilus* in China based on chromosome-level assembly and whole\-\genome sequencing data. Pest Manag. Sci..

[24] Sato K., Kadota Y., Gan P., Bino T., Uehara T., Yamaguchi K., Ichihashi Y., Maki N., Iwahori H., Suzuki T. (2018). High-quality genome sequence of the root-knot nematode *Meloidogyne arenaria* genotype A2-O. Genome Announc..

[25] Susič N., Koutsovoulos G.D., Riccio C., Danchin E.G., Blaxter M.L., Lunt D.H., Strajnar P., Širca S., Urek G., Stare B.G. (2020). Genome sequence of the root-knot nematode *Meloidogyne luci*. J. Nematol..

[26] Koutsovoulos G.D., Poullet M., Elashry A., Kozlowski D.K., Sallet E., Da Rocha M., Perfus-Barbeoch L., Martin-Jimenez C., Frey J.E., Ahrens C.H. (2020). Genome assembly and annotation of *Meloidogyne enterolobii*, an emerging parthenogenetic root-knot nematode. Sci. Data.

[27] Shinya R., Sun S., Dayi M., Tsai I.J., Miyama A., Chen A.F., Hasegawa K., Antoshechkin I., Kikuchi T., Sternberg P.W. (2022). Possible stochastic sex determination in *Bursaphelenchus* nematodes. Nat. Commun..

[28] Lawn D.A., Noel G.R. (1986). Gnotobiotic culture of *Pratylenchus scribneri* on carrot discs. Nematropica.

[29] Shen W., Le S., Li Y., Hu F. (2016). SeqKit: A cross-platform and ultrafast toolkit for FASTA/Q file manipulation. PLoS ONE.

[30] Cheng H., Concepcion G.T., Feng X., Zhang H., Li H. (2021). Haplotype-resolved de novo assembly using phased assembly graphs with hifiasm. Nat. Methods.

[31] Laetsch D.R., Blaxter M.L. (2017). BlobTools: Interrogation of genome assemblies. F1000Research.

[32] Li H. (2016). Minimap and miniasm: Fast mapping and de novo assembly for noisy long sequences. Bioinformatics.

[33] Li H., Handsaker B., Wysoker A., Fennell T., Ruan J., Homer N., Marth G., Abecasis G., Durbin R., 1000 Genome Project Data Processing Subgroup (2009). The Sequence alignment/map (SAM) format and SAMtools. Bioinformatics.

[34] Buchfink B., Reuter K., Drost H.G. (2021). Sensitive protein alignments at tree-of-life scale using DIAMOND. Nat. Methods.

[35] Camacho C., Coulouris G., Avagyan V., Ma N., Papadopoulos J., Bealer K., Madden T.L. (2008). BLAST+: Architecture and applications. BMC Bioinform..

[36] Guan D., McCarthy S.A., Wood J., Howe K., Wang Y., Durbin R. (2020). Identifying and removing haplotypic duplication in primary genome assemblies. Bioinformatics.

[37] Manni M., Berkeley M.R., Seppey M., Simão F.A., Zdobnov E.M. (2021). BUSCO update: Novel and streamlined workflows along with broader and deeper phylogenetic coverage for scoring of eukaryotic, prokaryotic, and viral genomes. Mol. Biol. Evol..

[38] Kokot M., Dlugosz M., Deorowicz S. (2017). KMC 3: Counting and manipulating k-mer statistics. Bioinformatics.

[39] Ranallo-Benavidez T.R., Jaron K.S., Schatz M.C. (2020). GenomeScope 2.0 and Smudgeplot for reference-free profiling of polyploid genomes. Nat. Commun..

[40] Bruna T., Hoff K.J., Lomsadze A., Stanke M., Borodovsky M. (2021). BRAKER2: Automatic eukaryotic genome annotation with genemark-ep+ and augustus supported by a protein database. NAR Genomic. Bioinform..

[41] Chen S., Zhou Y., Chen Y., Gu J. (2018). Fastp: An ultra-fast all-in-one FASTQ preprocessor. Bioinformatics.

[42] Dobin A., Davis C.A., Schlesinger F., Drenkow J., Zaleski C., Jha S., Batut P., Chaisson M., Gingeras T.R. (2013). STAR: Ultrafast universal RNA-seq aligner. Bioinformatics.

[43] Gabriel L., Hoff K.J., Brůna T., Borodovsky M., Stanke M. (2021). TSEBRA: Transcript selector for BRAKER. BMC Bioinform..

[44] Trapnell C., Williams B.A., Pertea G., Mortazavi A., Kwan G., Van Baren M.J., Salzberg S.L., Wold B.J., Pachter L. (2010). Transcript assembly and quantification by RNA-Seq reveals unannotated transcripts and isoform switching during cell differentiation. Nat. Biotech..

